# Food insecurity, food assistance, and physical and cognitive functioning among older Americans

**DOI:** 10.1371/journal.pone.0339720

**Published:** 2025-12-31

**Authors:** Usha Dhakal, Khalil El Asmar, Carlos F. Mendes deLeon

**Affiliations:** 1 Department of Oncology, Georgetown University School of Medicine, Washington, District of Columbia, United States of America; 2 Faculty of Health Sciences, American University of Beirut, Beirut, Lebanon; 3 Department of Global Health, Georgetown University, Washington, District of Columbia, United States of America; Lusofona University of Humanities and Technologies: Universidade Lusofona de Humanidades e Tecnologias, PORTUGAL

## Abstract

**Introduction:**

Food insecurity is associated with adverse late-life health outcomes, including disability, dementia, and mortality. But its role in changes in physical and cognitive functioning remains unclear, as does the impact of food assistance.

**Methods:**

We used weighted generalized estimating equations to investigate the association between food insecurity, food assistance, and physical and cognitive functioning among US adults aged 50 years and older (n = 6489). Using 2014 Health and Retirement Study (HRS) wave as the baseline, we extracted individual-level data from a HRS subsample from the 2013 Health Care and Nutrition Study and linked it to three additional HRS waves through 2020. This study included two outcome measures. First, physical functioning, a summary score of nine items that assessed difficulties in performing everyday physical activities (range 0–9), with higher scores indicating lower physical functioning. Second cognitive functioning, a composite score based on four cognitive tasks (range 0–27), with higher scores indicating better cognitive functioning.

**Results:**

In fully adjusted models, greater food insecurity was associated with lower physical functioning at baseline (β = 0.074 [95% CI 0.019, 0.129]) and showed marginal evidence of higher decline over time (0.010 [0.000, 0.020], *p* = .058). Food assistance was only associated with baseline physical functioning (−0·011 [−0·047, 0·025]). The triple interaction between food insecurity, food assistance, and time suggested that food assistance may attenuate declines in physical functioning (−0.014 [−0.030, 0.002], *p* = .078). Greater food insecurity (−0.206 [−0.305, −0.107]) and food assistance (−1.215 [−1.582, −0.848]) were associated with lower baseline cognitive functioning, but neither was associated with change over time. Food assistance did not moderate the association between food insecurity and changes in cognitive functioning over time.

**Conclusions:**

Food insecurity is associated with poorer physical and cognitive functioning in older adults. Strengthening policies and strategies that promote food assistance participation may help mitigate declines in physical functioning and improve health outcomes among older adults.

## Introduction

Food insecurity, defined as limited or uncertain access to adequate food [1], is common among older adults and associated with a variety of health risks [2]. In 2023, an estimated 18 million households, or 13.5% of all households in the U.S., reported food insecurity [3]. Recent data indicate a high prevalence of food insecurity among older adults as well, with an increasing trend over time [3–7]. Food insecurity affects almost one in ten (9.3%) of all households that contain an older adult (≥ 65 years) [3]. The prevalence is even higher (11.4%) in households of older adults living alone [3].

Food insecurity is associated with a range of adverse health outcomes, including chronic health conditions [8], disability [9], dementia [10,11], premature mortality [12], depression, and poor quality of life [13–16]. Emerging evidence suggests that food insecurity is associated with poor physical [8,17–19] and cognitive [20–22] functioning, which serve as manifestations of early stages of disability [8] and dementia outcomes [22], respectively. Even early stages of disability and dementia can result in significantly increased care needs of older adults. This underscores the importance of understanding the role of food insecurity in the progression of these declines with age, including the impact of food assistance.

Older adults in the U.S. often rely on multiple federal and other sources of food assistance when needed. Examples of federal food and nutrition assistance programs include the Supplemental Nutrition Assistance Program (SNAP), Meals on Wheels, Commodity Supplemental Food Program, and others [23,24]. SNAP is the largest assistance program used by older adults [23], with 7.2 million participants in 2022 [25]. In addition to federal programs, older adults also use other forms of food assistance. Examples include food banks/pantries, home-delivered meals, food drives, places of worship, community centers, and other sources to help fill their needs when they experience food insecurity or need to supplement their SNAP assistance. [26].

Although food assistance has largely been associated with health benefits for older adults, this evidence remains unclear. For example, participation in SNAP is associated with improved diabetes treatment adherence [27], slower decline in memory function [28], reduced hospitalizations, nursing home admissions [29], and healthcare spending [30], and reduced premature mortality [15]. But, other findings have failed to demonstrate protective effects of SNAP on cognition [10,31] and depression [16]. While these findings, although mixed, are largely indicative of positive health benefits for older adults, the degree to which participation in food assistance programs more generally, whether SNAP or other programs, confers such benefits, particularly in reducing the health risks associated with food insecurity, remains unclear.

The current study examines the association between food insecurity and changes in physical and cognitive functioning in a diverse cohort of US older adults. We also test the extent to which participation in food assistance programs mitigates the health risks associated with food insecurity.

## Materials and methods

### Study design and participants

Data for this study come from the publicly available RAND datasets of the Health and Retirement Study (HRS) and the HRS Health Care and Nutrition Study (HCNS). The HRS is an ongoing nationally representative biennial panel study of approximately 20,000 noninstitutionalized US adults 50 years and older [32]. The HCNS was conducted as a mail survey to a subsample of 12,418 HRS participants between the 2012 and 2014 HRS biannual waves and included questions on food security and food assistance. In this study, the 2013 HCNS food insecurity and assistance data were linked with the 2014 HRS wave, which was considered the baseline for the analysis. We included three additional biannual HRS waves (through 2020) to obtain follow-up data on the primary outcomes. To create our sample, we downloaded the publicly available deidentified data from the HRS website on November 20, 2024, and only included participants with non-missing data on all study variables and all five food insecurity items. Of the 8,073 participants who completed the HCNS survey, 6,742 had non-missing data on food insecurity, and 6,489 had non-missing data on all our study variables, constituting our final sample.

### Ethical approval

The HRS was conducted under the supervision of the University of Michigan Institutional Review Board, with ethics approval obtained and informed consent collected from all participants. Approval for this current study was granted exempt by Georgetown University Institutional Review Board. This study followed the Strengthening the Reporting of Observational Studies in Epidemiology (STROBE) reporting.

## Measures

### Outcomes: Physical functioning and cognitive functioning

Physical functioning was based on a composite score of nine items that assessed difficulties in performing the everyday physical activities (walking several blocks; walking one block; walking across the room; climbing several flights of stairs; climbing one flight of stairs; sitting for two hours; getting up from a chair; stooping or kneeling or crouching; and pushing or pulling a large object) [33]. The score ranged from 0–9, with higher scores indicating lower physical functioning. Cognitive functioning was based on a composite score of cognitive tasks (immediate and delayed word recall, the serial seven subtraction test, and backwards count from 20), yielding a range from 0–27 and higher scores indicating better cognitive functioning [34].

### Predictors: Food insecurity and food assistance

Food insecurity was measured based on a standardized instrument, Six-Item Short Form Food Security Survey Module (USDA FSSM), which assesses household food access and consumption during the last 12 months [35,36]. In HCNS, participants were asked to think about their household food consumption in the past twelve months and respond to those items. The first two items included: “The food that we bought just didn’t last and we didn’t have enough money to get more”, and “We couldn’t afford to eat balanced meals”. The next items assessed if the respondent or other adults in the household ever cut the size or skip meals because there wasn’t enough money for food and its frequency. The last two items assessed whether the respondent ever ate less than they felt they should or they were ever hungry but didn’t eat because there wasn’t enough money for food. Following the U.S. Household Food Security Survey Module: Six-Item Short Form Economic Research Service (USDA FSSM) scoring guidelines [36], we summed the responses into a total food insecurity score (range 0–6), with higher scores indicating more food insecurity. Food assistance items in HCNS assessed whether respondents received any free or subsidized food from six sources: food bank or food pantry, church, shelter, meals on wheels, senior brown-bag or other home delivered meal service, and other source of food donations in the last twelve months. Use of food assistance (yes/no) was defined based on the report of using at least one food assistance source. Both food insecurity and food assistance were time invariant variables and only measured once.

### Covariates

We included control variables for multiple demographic, socio-economic, and health factors. Demographic factors included *age in years* (centered around mean), *gender* (male/female), and *race/ethnicity* (Non-Hispanic White, Non-Hispanic Black, Non-Hispanic Other, and Hispanic). Socio-economic factors included *years of education* (years of completed schooling) and *wealth* (quartiles of net wealth, based on assessment of the total value of all assets and debts). Health factors included *number of chronic conditions* (sum of 8 physician-diagnosed self-reported chronic health conditions: high blood pressure, diabetes, cancer, lung disease, heart disease, stroke, psychiatric problems, and arthritis and *number of depressive symptoms* (based on the 8-item Center for Epidemiologic Studies Depression Scale) [16]. Follow-up *time* was computed by subtracting the respondents’ baseline interview date from the corresponding follow-up interview dates. Resulting values represent the number of years since baseline, i.e., 2014.

### Statistical analysis

We summarized participants’ characteristics by performing descriptive statistics; mean and standard deviation for continuous variables and frequency and percentage for categorical variables. We used a series of longitudinal regression models to test the associations between food insecurity, food assistance, and the physical and cognitive functioning outcome variables, which included sequential adjustments for potential confounders. In the first model, we tested the association between food insecurity and each outcome after adjusting for demographic and socio-economic variables (age, gender, race/ethnicity, net wealth, and education). This model also included a term for the interaction between food insecurity and time since baseline to test whether food insecurity was associated with change in each outcome during follow-up. In the second model, we added terms for food assistance and its interaction with time, followed by a triple interaction term for food insecurity, food assistance and time in the third model, to test whether food assistance modified the association between food insecurity and changes in each outcome during follow-up. The final, fully adjusted model included additional control for the number of chronic health conditions and depressive symptoms. All models were fitted using generalized estimating equations, adjusted for the HCNS sampling weight, and were inverse-probability weighted to account for attrition during follow-up. An alpha level of 0.05 was used to indicate statistical significance. All analyses were conducted using SAS® V9.4.

## Results

There were 6489 participants at baseline, with an average age of 67 years (Table 1). Just over half of the participants were female (54.9%) and almost four-fifths were Non-Hispanic White (79.2%). On average, participants had approximately 13 years of education, and a net wealth of 557902 US dollars, and reported having two chronic conditions ($\overset{―}{X}$ = 2.1) and one depressive symptom ($\overset{―}{X}$= 1.3). In the past 12 months, 1152 participants (16.6%) had experienced low/very low food security, defined as having a food insecurity score of 2 and higher. Similarly, 1105 participants (17.0%) reported using food assistance during the same time. Although the sample characteristics for high/marginal and low/very low food security groups followed a similar pattern compared to the overall sample (Table 1), contrastingly, among the 1152 participants who had low/very low food security, the average age was lower ($\overset{―}{X}$ = 63.3), there were more females (60.5%), and less Non-Hispanic Whites (53.2%). They also had fewer years of education ($\overset{―}{X}$ = 11.7), lower net wealth ($\overset{―}{X}$ = 71191), greater number of depressive symptoms ($\overset{―}{X}$ = 2.7), and more food assistance (46.9%) users.

**Table 1:** Characteristics of baseline sample (2014), overall and by food security status.

Sample Characteristics	Total sample (n = 6489)	Food security status	
		**High/marginal** **(n = 5337)**	**Low/very low (n = 1152)**
Age, $\overset{―}{X}$	67·0 (0·3)	67·7 (0·3)	63·3 (0·4)
Gender, %			
Female	3818 (54·9%)	3085 (53·8%)	733 (60·5%)
Male	2671 (45·1%)	2252 (46·2%)	419 (39·5%)
Race/ethnicity, %			
Non-Hispanic White	4534 (79·2%)	4064 (84·3%)	470 (53·2%)
Non-Hispanic Black	1048 (9·6%)	711 (7·6%)	337 (19·5%)
Non-Hispanic Other	198 (3·3%)	142 (2·7%)	56 (6·6%)
Hispanic	709 (7·9%)	420 (5·4%)	289 (20·7%)
Education, years, $\overset{―}{X}$	13·3 (0·1)	13·6 (0·1)	11·7 (0·2)
Net wealth (USD), $\overset{―}{X}$	557646 (35897)	654735 (41213)	71191 (12773)
Food assistance, %			
Yes	1105 (14·3%)	531 (7·8%)	574 (46·9%)
No	5384 (85·7%)	4806 (92·2%)	578 (53·1%)
Number of chronic conditions, $\overset{―}{X}$	2·1 (0·0)	2·0 (0·0)	2·6 (0·1)
Number of depressive symptoms, $\overset{―}{X}$	1·3 (0·0)	1·1 (0·0)	2·7 (0·1)

Data shown are n (%) or mean (SE); $\overset{―}{X}$ = mean; % = percentage; SE = standard error; means and percentages are weighted; frequencies are unweighted. High/marginal food security comprises of participants with food insecurity scores of 0 and 1. Low/very low food security comprises of participants with food insecurity scores of 2–6, indicating greater food insecurity.

### Findings for physical functioning

In all models (Table 2), greater food insecurity was significantly associated with higher physical functioning scores, indicating poorer physical functioning. At baseline (time = 0), a one unit increase in food insecurity score was associated with a 0.292 higher physical functioning score (β = 0.292 [0.241, 0.343]) in the minimally adjusted model (model 1). The interaction between food insecurity and time was not significant (β = −0·002 [−0·009, 0·005], indicating it was not associated with greater increases in physical functioning scores (more decline) over time. After adjusting for food assistance and its interaction with time (model 2), the association persisted, although the main effect for food insecurity was somewhat reduced (β = 0.240 [0.186, 0.294]). At baseline, food assistance was significantly associated with higher physical functioning scores (β = 0.617 [0.412,0.822]), and with increasingly lower physical functioning scores over time (β = −0.034 [−0.065, −0.004]), indicating less decline. The triple interaction term between food assistance, food insecurity, and time (model 3) was not significant (β = −0.012 [−0.028, 0.004]).

**Table 2:** Associations between food insecurity, food assistance, and change in physical functioning.

	Model 1	Model 2	Model 3	Model 4
Intercept	3·949 (3·622, 4·277)^***^	3·814 (3·485, 4·143)^***^	3·814 (3·485, 4·143)^***^	1·797 (1·500, 2·095)^***^
Age	0·058 (0·049, 0·066)^***^	0·057 (0·049, 0·066)^***^	0·057 (0·049, 0·066)^***^	0·021 (0·013, 0·029)^***^
Age*age	0·001 (0·000, 0·001)^*^	0·001 (0·000, 0·001)^*^	0·001 (0·000, 0·001)	0·001 (0·001, 0·002)^***^
Female	−0·405 (−0·508, −0·301)^***^	−0·405 (−0·507, −0·302)^***^	−0·405 (−0·507, −0·302)^***^	−0·375 (−0·463, −0·287)^***^
Hispanic	−0·405 (−0·603, −0·206)^***^	−0·408 (−0·606, −0·211)^***^	−0·412 (−0·610, −0·214)^***^	−0·222 (−0·394, −0·050)
Non-Hispanic Black	0·058 (−0·123, 0·239)	−0·043 (−0·226, 0·140)	−0·045 (−0·229, 0·138)	−0·034 (−0·192, 0·124)
Non-Hispanic Others	−0·205 (−0·481, 0·071)	−0·222 (−0·497, 0·050)	−0·224 (−0·497, 0·048)	−0·137 (−0·362, 0·089)
Time	−0·001 (−0·028, 0·026)	0·003 (−0·024, 0·030)	0·001 (−0·026, 0·028)	0·047 (0·020, 0·073)^***^
Time*time	0·006 (0·002, 0·010)^**^	0·005 (0·001, 0·010)^*^	0·005 (0·001, 0·010)^*^	−0·001 (−0·005, 0·004)
Age×time	0·006 (0·005, 0·007)^***^	0·006 (0·005, 0·007)^***^	0·006 (0·005, 0·007)^***^	0·006 (0·005, 0·007)^***^
Quartiles of net wealth	−0·452 (−0·508, −0·396)^***^	−0·428 (−0·484, −0·371)^***^	−0·427 (−0·484, −0·370)^***^	−0·269 (−0·318, −0·219)^***^
Years of education	−0·087 (−0·109, −0·066)^***^	−0·083 (−0·105, −0·061)^***^	−0·083 (−0·105, −0·061)^***^	−0·060(−0·079, −0·041)^***^
Sampling weight	−0·001 (−0·008, 0·005)	−0·001 (−0·008, 0·005)	−0·001 (−0·008, 0·005)	0·001 (−0·005, 0·007)
Food insecurity	0·292 (0·241, 0·343)^***^	0·240 (0·186, 0·294)^***^	0·234 (0·164, 0·303)^***^	0·074 (0·019, 0·129)^***^
Food insecurity×time	−0·002 (−0·009, 0·005)	0·002 (−0·006, 0·010)	0·006 (−0·004, 0·017)	0·010 (0·000, 0·020)
Food assistance		0·617 (0·412, 0·822)^***^	0·599 (0·350, 0·847)^***^	0·379 (0·167, 0·590)^***^
Food assistance×time		−0·034 (−0·065, −0·004)^*^	−0·016 (−0·053, 0·021)	−0·011 (−0·047, 0·025)
Food insecurity×food assistance			0·015 (−0·091, 0·121)	0·021 (−0·066, 0·109)
Food insecurity×food assistance×time			−0·012 (−0·028, 0·004)	−0·014 (−0·030, 0·002)
Number of depressive symptoms				0·313 (0·282, 0·343)^***^
Number of chronic conditions				0·560 (0·525, 0·596)^***^

Data shown are β [95% CI]. β = parameter estimates; CI = confidence interval; Ref = reference; NHW = Non-Hispanic White. Higher physical functioning score indicates lower physical functioning. ^*^p < .05, ^**^p < .01, ^***^p < .001

In the fully adjusted model (model 4), greater food insecurity remained significantly associated with lower physical functioning at baseline (β = 0.074 [0.019, 0.129]). This model also showed a marginally significant association between food insecurity and time (β = 0.010 [0.000, 0.020], *p* = 0.058), suggesting that greater food insecurity is associated with increasingly higher physical functioning scores (more decline) over time. In this model, the triple interaction effect between food assistance, food insecurity, and time (β = −0.014 [−0.030, 0.002], *p* = 0.078) suggests the possibility that food assistance reduces the association between greater food insecurity and increases in physical functioning scores (i.e., less decline) over time, while acknowledging that this effect failed to reach statistical significance.

To interpret the public health relevance of these findings, we computed the difference in predicted physical functioning scores for food insecurity scores of 0 (10^th^ percentile) and 3 (90^th^ percentile) in the fully adjusted model and compared that difference with the size of the effect of aging (coefficient for time β = 0.047) on physical functioning scores. The 90^th^-10^th^ percentile difference in food insecurity scores was associated with a 0.222 (i.e., 3 × 0.074) point higher physical functioning score (poorer physical functioning) among older adults not receiving food assistance, holding all other variables constant. This difference is comparable to approximately 5 years of average decline in physical functioning (5 × 0.047 = 0.235). For older adults receiving food assistance, the effect of food insecurity is best represented by the main effect of food insecurity (β = 0.074) plus the interaction term between food insecurity and food assistance (β = 0.021; note, this estimate was not significant). Together, they add up to a 0.285 (i.e., 3 × 0.095) point higher score in physical functioning for those at the 90^th^ percentile versus the 10^th^ percentile of the food insecurity measure. This difference is comparable to a decline of over approximately 6 years of average decline (6 × 0.047 = 0.282), slightly and not significantly more than the main effect for food insecurity.

### Findings for cognitive functioning

In all models (Table 3), greater food insecurity was significantly associated with lower cognitive functioning. At baseline (time = 0), a one unit increase in food insecurity score was associated with a 0.294 lower cognitive functioning score (β = −0.294 [−0.366, −0.223]) in the minimally adjusted model (model 1). The interaction between food insecurity and time was not significant (β = 0·008 [−0·005, 0·022], indicating it was not associated with change in cognitive functioning scores over time. After adjusting for food assistance and its interaction with time, the association between food insecurity and cognitive functioning remained significant (model 2), although the coefficient for the main effect of food insecurity was reduced by about 27% (β = −0.207 [−0.283, −0.131]). At baseline, food assistance was significantly associated with lower cognitive functioning scores (β = −1·026 (−1·327, −0·726)] and with a marginally significant association with less decline in cognitive functioning over time (β = 0.057 [−0.007, 0.121], *p* = 0.081). After adding the triple interaction term (model 3), associations for food insecurity and food assistance remained mostly unchanged. In addition, the interaction between food insecurity and food assistance was significant (β = 0.178 [0.032, 0.324]), indicating that food assistance reduced the negative association between food insecurity and cognitive functioning. The triple interaction between food assistance, food insecurity, and time was not significant (β = −0.009 [−0.040, 0.023]), indicating that food assistance did not moderate the relationship between food insecurity and cognitive decline over time.

**Table 3:** Associations between food insecurity, food assistance, and change in cognitive functioning.

	Model 1	Model 2	Model 3	Model 4
Intercept	11·444 (10·906, 11·982)^***^	11·665 (11·125, 12·205)^***^	11·733 (11·192, 12·273)^***^	12·495 (11·922, 13·067)^***^
Age	−0·101 (−0·115, −0·087)^***^	−0·100 (−0·114, −0·086)^***^	−0·100 (−0·114, −0·086)^***^	−0·089 (−0·103, −0·074)*^**^
Age*age	−0·004 (−0·005, −0·003)^***^	−0·004 (−0·005, −0·003)^***^	−0·004 (−0·005, −0·003)^***^	−0·004 (−0·005, −0·003)^***^
Female	−0·866 (−1·029, −0·702)^***^	−0·865 (−1·028, −0·702)^***^	−0·869 (−1·031, −0·706)^***^	−0·895 (−1·056, −0·734)^***^
Hispanic	−0·419 (−0·734, −0·103)	−0·411 (−0·725, −0·098)	−0·387 (−0·699, −0·075)	−0·429 (−0·741, −0·118)
Non-Hispanic Black	−1·895 (−2·161, −1·63)^***^	−1·727 (−1·996, −1·458)^***^	−1·709 (−1·978, −1·440)^***^	−1·711 (−1·976, −1·446)^***^
Non-Hispanic Others	−1·178 (−1·653, −0·704)^***^	−1·15 (−1·623, −0·678)^***^	−1·125 (−1·600, −0·650)^***^	−1·159 (−1·626, −0·692)^***^
Time	−0·002 (−0·060, 0·057)	−0·009 (−0·068, 0·049)	−0·011 (−0·070, 0·048)	−0·033 (−0·092, 0·027)
Time*time	−0·004 (−0·013, 0·006)	−0·004 (−0·013, 0·006)	−0·003 (−0·013, 0·006)	−0·001 (−0·010, 0·009)
Age×time	−0·011 (−0·013, −0·009)^***^	−0·011 (−0·013, −0·009)^***^	−0·011 (−0·013, −0·009)^***^	−0·011 (−0·013, −0·009)^***^
Quartiles of net wealth	0·364 (0·275, 0·452)^***^	0·324 (0·234, 0·413)^***^	0·312 (0·222, 0·402)^***^	0·254 (0·164, 0·345)^***^
Years of education	0·401 (0·364, 0·438)^***^	0·394 (0·357, 0·431)^***^	0·392 (0·355, 0·429)^***^	0·382 (0·345, 0·418)^***^
Sampling weight	−0·008 (−0·019, 0·003)	−0·008 (−0·019, 0·002)	−0·008 (−0·018, 0·003)	−0·008 (−0·019, 0·002)
Food insecurity	−0·294 (−0·366, −0·223)^***^	−0·207 (−0·283, −0·131)^***^	−0·281 (−0·381, −0·182)^***^	−0·206 (−0·305, −0·107)^***^
Food insecurity×time	0·008 (−0·005, 0·022)	0·002 (−0·014, 0·018)	0·006 (−0·015, 0·026)	0·004 (−0·017, 0·024)
Food assistance		−1·026 (−1·327, −0·726)^***^	−1·309 (−1·679, −0·940)^***^	−1·215 (−1·582, −0·848)^***^
Food assistance×time		0·057 (−0·007, 0·121)	0·068 (−0·012, 0·147)	0·064 (−0·016, 0·143)
Food insecurity×food assistance			0·178 (0·032, 0·324)	0·175 (0·030, 0·319)^*^
Food insecurity×food assistance×time			−0·009 (−0·040, 0·023)	−0·007 (−0·038, 0·024)
Number of depressive symptoms				−0·177 (−0·223, −0·132)^***^
Number of chronic conditions				−0·174 (−0·237, −0·111)^***^

Data shown are β [95% CI]. β = parameter estimates; CI = confidence interval; Ref = reference; NHW = Non-Hispanic White. Higher cognitive functioning score indicates better cognitive functioning. ^*^p < .05, ^**^p < .01, ^***^p < .001

In the fully adjusted model (model 4), associations remained mostly unchanged. Greater food insecurity remained significantly associated with lower cognitive functioning at baseline (β = −0.206 [−0.305, −0.107]) but not with change in cognitive functioning over time (β = 0.004 [−0.017, 0.024]). Similarly, food assistance remained significantly associated with lower cognitive functioning at baseline (β = −1·215 [−1·582, −0·848]) but not with change over time (β = 0·064 [−0·016, 0·143]). The interaction between food insecurity and food assistance (β = 0.175 [0.030, 0.319] remained significant as well in this model. The triple interaction between food assistance, food insecurity, and time was still not significant (β = −0.174 [−0.237, −0.111]).

To interpret the public health relevance of these findings, we computed the difference in predicted cognitive functioning scores for food insecurity scores of 0 (10^th^ percentile) and 3 (90^th^ percentile) in the fully adjusted model and compared that difference with the size of the effect of aging (coefficient for time β = −0.033) on cognitive functioning scores. The 90^th^-10^th^ percentile difference in food insecurity scores was associated with a 0.618 (i.e., 3 × −0.206 = −0.618) point lower cognitive functioning score (poorer cognitive functioning) among older adults not receiving food assistance, holding all other variables constant. This difference is comparable to approximately 19 years of decline (19 × −0.033 = −0.627). For older adults receiving food assistance, the effect of food insecurity is represented by the main effect of food insecurity (β = −0.206) plus the interaction term between food insecurity and food assistance (β = 0.175), which add up to a 0.093 (i.e., 3 × −0.031) point lower score in cognitive functioning for those at the 90^th^ percentile versus the 10^th^ percentile of the food insecurity measure. This difference is comparable to a decline of approximately 3 years of aging (3 × −0.033 = −0.099). These comparisons indicate that food insecurity is associated with substantially lower cognitive functioning but this association is substantially attenuated for older adults receiving food assistance.

## Discussion

Our findings from a large and diverse sample of older adults indicate that those who report greater food insecurity or use food assistance exhibit lower levels of physical and cognitive functioning. Among those who did not use food assistance, we found a marginally significant association between greater food insecurity and greater decline in physical functioning over time, while there was also a suggestion that this association is mitigated by the use of food assistance. Neither food insecurity nor use of food assistance were associated with changes in cognitive functioning over time. These findings suggest that food insecurity may play a contributing role to poorer physical and cognitive functioning outcomes in older adults and raise the possibility that food assistance might alleviate these health risks, at least for declines in physical functioning.

Our findings regarding physical functioning are generally in line with those reported previously [8,17–19], even if most studies have only focused on the cross-sectional relationship between food insecurity and physical functioning. Our work adds an important new aspect to this relationship; we found a longitudinal association suggestive of a prospective role of food insecurity in worsening physical functioning for older adults, especially for those not using food assistance. Our results further suggest that food assistance may mitigate this effect. The beneficial effect of food assistance was significantly observed among males and Non-Hispanic Whites. Given the potential benefit of food assistance programs in alleviating the physical health risks of food insecurity, this finding warrants more detailed investigation in future studies and intervention trials. Additionally, food insecurity often co-exist with other social risk factors, such as poverty, lack of housing, transportation, and healthcare access, future studies should also consider the impact of these factors [37,38].

Our findings appear inconsistent with those from previous studies, including those using HRS data [11,21] which have generally found reliable evidence for an association between food insecurity and decline in cognitive functioning [11,20,21] and dementia risk [10,11]. Our data suggest only a cross-sectional relationship, which leaves the direction of the underlying causal mechanism uncertain. However, there are notable differences between our own study and others that may account for the inconsistency in findings. First, we used a well-validated measure of food insecurity while others have relied on 2- or 5-item screening questions [20,21]. Second, we focused on a global measure of cognitive functioning while others have found the association between food insecurity and cognitive decline restricted to declines in either memory [11,20,28] or executive functioning [20]. Finally, our findings are not necessarily at odds with those related to dementia risk: older adults with food insecurity in our study had significantly poorer cognitive functioning and therefore likely to reach the threshold for a dementia diagnosis sooner than those without food insecurity. The mixed pattern of findings in this literature suggests that evidence for a causal effect of food insecurity on cognitive decline and dementia risk remains unsettled.

Several mechanisms may explain our findings regarding the possible contribution of food insecurity in worsening physical functioning. Food insecurity often leads to poor access to nutritious food and compromised diet quality [39], which may impact functional limitations through several pathways due to poor nutrition [2,40]^.^ First, poor nutrition may lower energy levels [40], which in turn may lead to greater difficulty in performing basic physical tasks and activities^2^. Second, poor nutritious food intake can result in deficiency of required vitamins, minerals, and hormones, which may contribute to chronic stress and inflammation [2]. In turn, chronic stress and inflammation can exacerbate chronic conditions and their effect on functional limitations [2,41], resulting in incident disability and premature mortality [42].

Few studies to date have examined the potential benefits of food assistance in the context of food insecurity and aging-related health outcomes in older adults. Initial findings suggest that food assistance, in particular through the SNAP program in the US, may provide a benefit with respect to cognitive functioning [31] and dementia risk [10,11] even if the overall evidence remains mixed [28]. The unique contribution of this study is that we considered several forms of food assistance, unlike other studies that only assessed the federal SNAP program, as many older adults seek this assistance from a variety of public and private sources [26]. In addition, we also specifically focused on the mitigating effect of food assistance, instead of its main effect, to alleviate declines in physical and cognitive functioning due to food insecurity. While we found no evidence for such a mitigating effect for cognitive decline, our data suggest the possibility that food assistance reduces the impact of food insecurity on changes in physical functioning.

Regardless of the directionality of the relationship between food insecurity and physical or cognitive health outcomes in older adults, our findings offer some initial insights into possible interventions and social policies that may not only alleviate the condition of food insecurity itself, but may have the added benefit of reducing downstream health risks. First, social programs such as Meals on Wheels that promote food access among food-insecure older adults may ease their immediate nutritional deficits and provide longer-term health benefits, especially with regard to markers of physical health. Second, our findings may motivate health care practitioners and other service providers to screen for food insecurity [37,38] and provide food-insecure older adults and their family members with information on formal food assistance programs, such as Meals on Wheels and home-delivered meals [37], as well as local volunteer food assistance services offered through churches or other places of worship, food banks/pantries, or other community-based food resources [43,44]. Finally, it may be important to reduce stigma associated with either food insecurity or use of food assistance in both the clinical and public health arenas [37].

Our study has notable limitations. HCNS was only administered once, which limited our ability to determine the chronicity of food insecurity and participation in food assistance resources. We also relied on relatively brief, global measures of physical and cognitive functioning, although there is currently little evidence to suggest that food insecurity or food assistance should affect only specific domains within each of these functional outcomes. Last, we used only four waves of data spanning a total of 6 years of follow-up, even though aging-related declines in physical and cognitive functions tend to develop over much longer time periods. The strength of these findings includes the use of a diverse sample of older adults with up to six years of follow-up data and the use of a well-validated and widely used measure of food insecurity. Another strength is that we considered food assistance that included the use of both formal programs and local plans relying on volunteer contributions.

## Conclusions

To summarize, we found that in US older adults, food insecurity is associated with poorer physical and cognitive functioning, although it did not seem to be related to changes in these outcomes over time. We also found some evidence that food assistance may act as a buffer to compensate for the decline in physical functioning, but not cognitive functioning, among those who experienced food insecurity. Our findings suggest the importance of food assistance programs, either through federal or state policies or by private organizations in supporting functional health among older adults experiencing food insecurity, thereby potentially reducing the risk of disability onset in this vulnerable population. Additionally, making community initiatives such as local food banks and food pantries available for all individuals regardless of their income could increase the accessibility to food and reduce the stigma associated with participation in food assistance programs. Additional research should confirm these findings to inform aging policies, including increasing food access through the use of both public and private food assistance programs for older adults.
